# Useful Test for Classification of Cerebral Infarction at Hospital Specializing in Neurosurgery

**DOI:** 10.3400/avd.oa.22-00099

**Published:** 2022-12-25

**Authors:** Hirotaka Yoshida, Kazutoshi Nishitani

**Affiliations:** 1 Department of Neurosurgery, Tokyo General Hospital, Tokyo, Japan; 2 Department of Neurosurgery, Kitahara International Hospital, Tokyo, Japan

**Keywords:** cryptogenic stroke, insertable cardiac monitor (ICM), BNP, paroxysmal atrial fibrillation (PAF), mechanical thrombectomy

## Abstract

**Background and Purpose:** There are many cases of cerebral infarction of unknown etiology in which the embolic sources cannot be identified including atrial fibrillation despite achievement of complete revascularization after thrombectomy.

**Method:** An analysis was conducted for 556 consecutive cases of patients who were hospitalized for cerebral infarction to determine the significance of accurate classification of disease type and investigation into causes of cerebral infarction of unknown cause.

**Result:** According to the Trials of Org 10172 in Acute Stroke Treatment (TOAST) classification, cerebral infarction of other/unknown etiology was observed in 94 cases, of which 22 cases were found to have causes by additional workup. Implantable cardiac monitors were inserted in 15 of 76 cases of cryptogenic cerebral infarction, of which 4 cases (26%) showed detection of paroxysmal atrial fibrillation (PAF) during observation period (223–384 days).

**Conclusion:** Brain natriuretic peptide (BNP) measurement, abdomen-pelvic computed tomography (CT), cardiac monitoring for 1 week, and implantable cardiac monitors (ICM) were useful for the classification of disease type and detection of cryptogenic atrial fibrillation. (This is secondary publication from J Jpn Coll Angiol 2021; 61: 49–55.)

## Introduction

Mechanical thrombectomy has been established and is widely used as an effective treatment for acute cerebral infarction caused by major arterial occlusion. In some cases, disease subtypes are difficult to determine because atrial fibrillation (AF) is not found at the time of diagnosis and no arteriosclerotic changes in major arteries are identified despite successful complete revascularization. The Trial of Org 10172 in Acute Stroke Treatment (TOAST) classification system is generally used to classify cerebral infarction subtypes. Accurate subtype classification is critical because distinct treatment strategies are used for secondary prevention depending on the subtype. However, the accuracy of exploration for the etiology of the identified subtypes differs substantially from one hospital to another. Recently, cerebral infarction caused by unidentifiable etiologies despite thorough examination has been defined as cryptogenic stroke, which remains largely unclear in terms of features and pathophysiology. Causes of cryptogenic stroke include non-embolic pathological conditions as well as embolic stroke of undetermined source (ESUS), which accounts for the majority. Identification of tests useful for subtyping of cerebral infarction is needed.

## Subjects and Methods

Of 556 new cases of cerebral infarction treated on inpatient basis at Medical Corporation KNI Kitahara International Hospital between April 1, 2018 and March 30, 2019, 7 were excluded owing to the lack of data trackability, and 549 (312 men and 237 women; mean age, 76.2±11.9 years) were included in this study.

**Figure 1:**
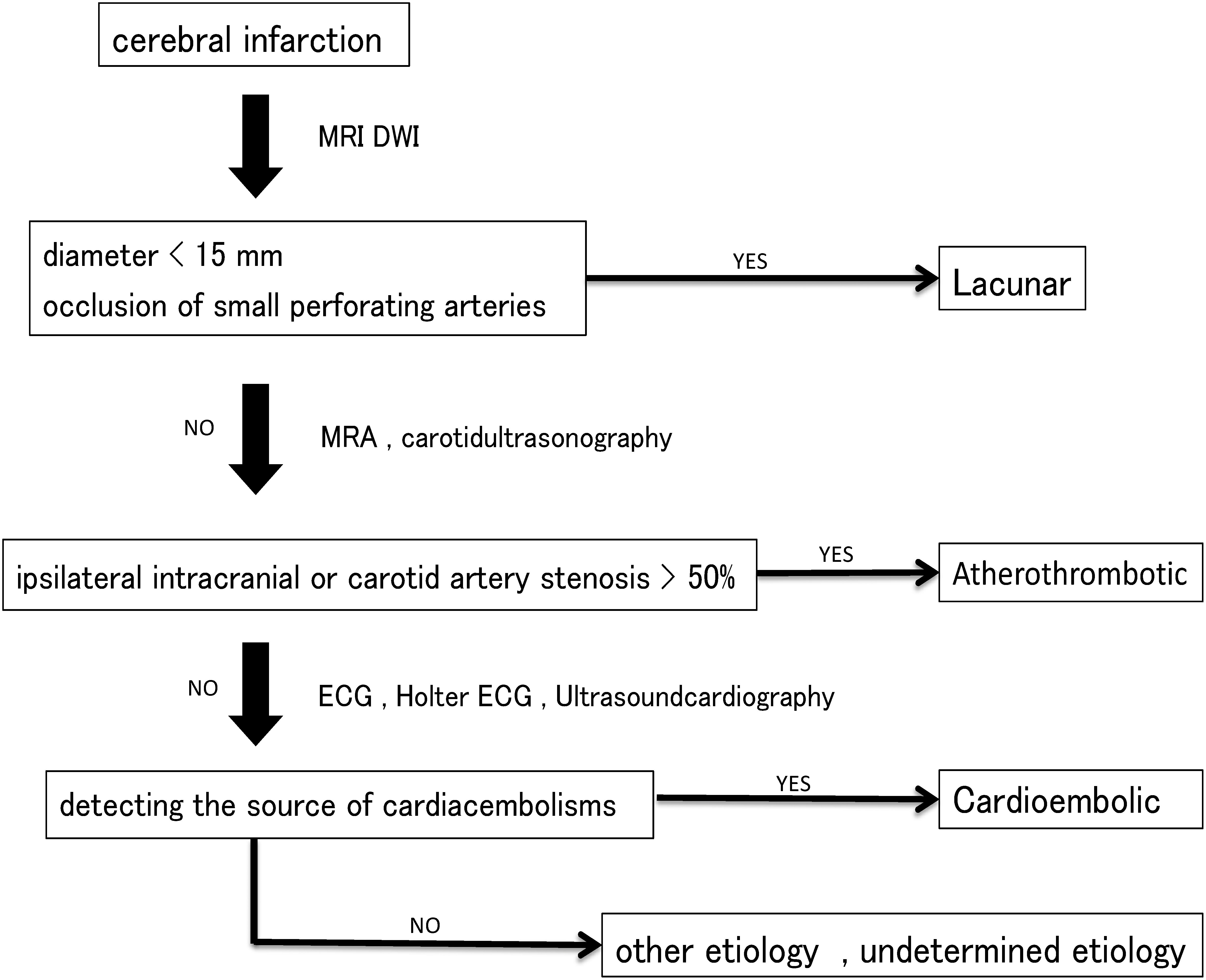
Fig. 1 Subtype of acute ischemic stroke, using the TOAST classification.

The subtypes were determined by neurosurgeons and stroke specialists according to the TOAST classification system at the time of admission to and discharge from the hospital (**Fig. 1**), and the male/female ratio, mean age, brain natriuretic peptide (BNP), and HbA1c in cases of different subtypes were evaluated to find clinical characteristics. The Tukey–Kramer honestly significant difference (HSD) test was used to compare intergroup BNP levels.

Patients with cerebral infarction categorized into “other determined etiology/undetermined etiology” according to the TOAST classification underwent special blood tests (thrombohemostatic blood tests) and thoracic-abdominal-pelvic computed tomography (CT) in addition to Holter monitoring and transthoracic echocardiographic examinations.

Special blood test items were as follows: protein C activity, protein S activity, anticardiolipin antibody, antinuclear antibody, total homocysteine, vitamin B12, folic acid, and soluble IL-2 receptor levels. Tumor markers measured were as follows: α-fetoprotein, carcinoembryonic antigen, carbohydrate antigen 19-9, Pro gastrin-releasing peptide, and squamous cell carcinoma antigen. Patients with elevated levels of the tumor markers and D-dimer underwent contrast-enhanced CT examinations. During contrast-enhanced thoracic-abdominal-pelvic CT examinations, patients also underwent 3D-CT angiography for evaluation of aortic arch plaques. All patients with elevated D-dimer levels underwent venous ultrasonography of lower extremities for the detection of deep venous thrombosis (DVT).

In cases of cryptogenic stroke, for the etiologies that remained unidentified after these examinations, insertable cardiac monitors (ICMs) were proactively used to detect paroxysmal atrial fibrillation (PAF). The medical records were then reviewed retrospectively to evaluate the usefulness of each test.

This study was approved by the Institutional Review Board of Kitahara International Hospital (approval number, 60; approved on March 23, 2019).

## Results

At the time of hospitalization, the cerebral infarction subtypes based on the TOAST classifications were large-artery atherosclerotic stroke in 143 cases (26%), lacunar infarction in 199 cases (36%), cardioembolic stroke in 113 cases (21%), and cerebral infarction of other determined etiology/undetermined etiology in 94 cases (17%). Of patients with cardioembolic stroke, 86 had confirmed AF or PAF at the time of hospitalization, and PAF was first detected after hospitalization in 25 cases. On average, PAF was detected 6.54 days (2–77 days; median 3.0 days) after hospitalization.

Regarding patient background, 57% of the subjects were men and and 43% women. In terms of the disease subtypes, large-artery atherosclerotic stroke was clearly more common among men; in addition, lacunar infarction and cerebral infarction of other determined etiology were more common among men. Cardioembolic stroke was the only subtype that appeared to occur more commonly among women. The HbA1c level did not differ among the disease subtypes. The mean BNP level at the time of hospitalization was 40.4 pg/mL among patients with large-artery atherosclerotic stroke, 40.4 pg/mL among patients with lacunar infarction, 226.9 pg/mL among patients with cardioembolic stroke, and 58.5 pg/mL among patients with cerebral infarction of other determined and undetermined etiologies (patient background data are summarized in **Table 1**).

**Table 1:** Table 1 Baseline characteristics of patients with acute ischemic stroke

	Cardioembolic	Atherothrombotic	Lacunar	Other	*P* value
n	111	141	196	104	
Age, median (IQR)	82 (76–89)	78 (69–85)	75 (67–82)	77 (67.3–85)	<0.0001^1^
Female, median (IQR)	61 (55.0%)	47 (33.3%)	82 (41.8%)	49 (47.1%)	0.006^2^
HbA1c, median (IQR)	5.8 (5.6–6.2)	6.1 (5.6–6.8)	5.9 (5.6–6.5)	5.8 (5.5–6.3)	0.02^1^
BNP, median (IQR)	226.9 (137.6–402.2)	40.4 (19.6–112.9)	40.4 (20.8–84.3)	58.5 (22.7–135.1)	<0.0001^1^

^1^ Kruskal–Wallis test, ^2^ χ^2^ test

**Figure 2:**
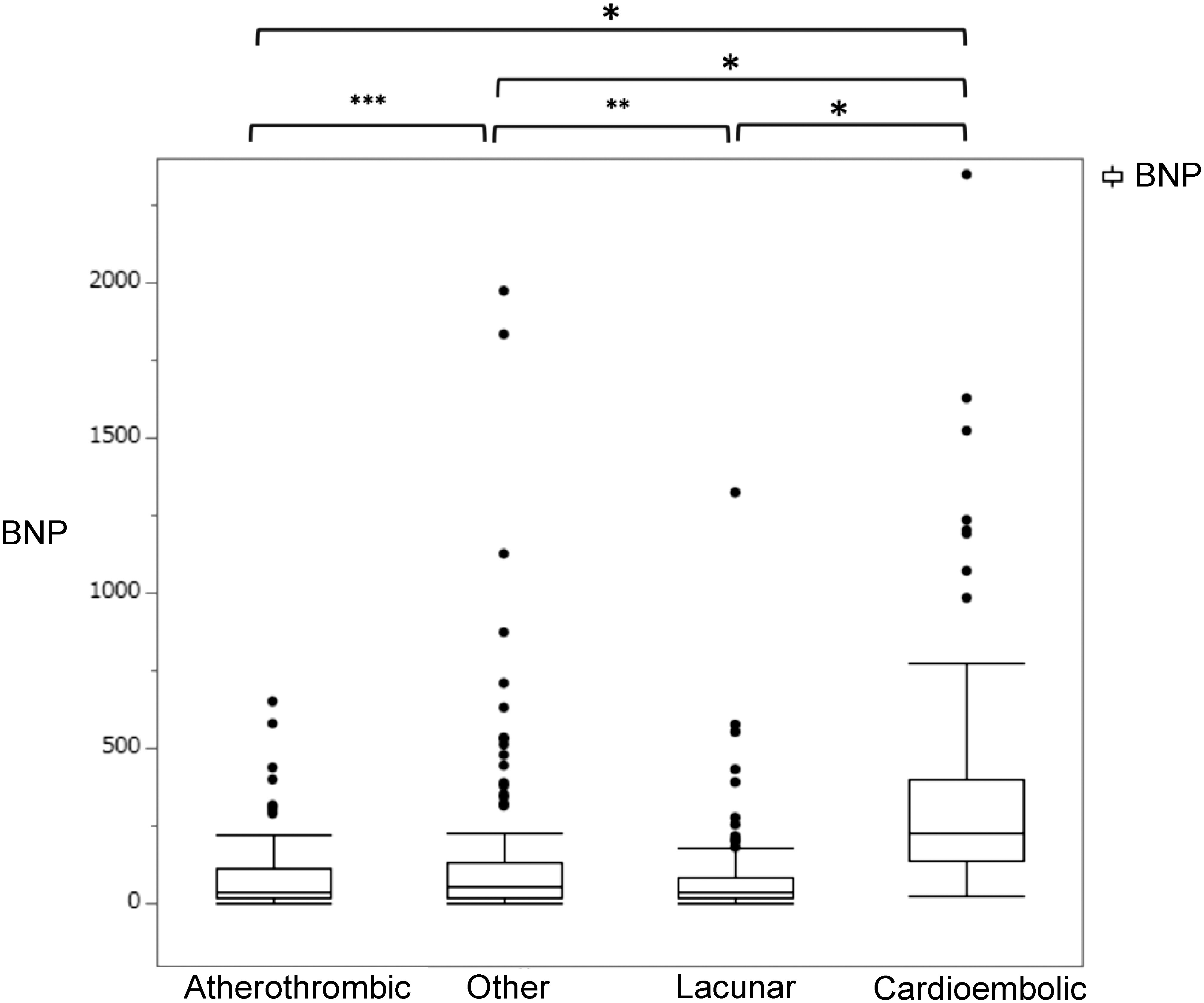
Fig. 2 Brain natriuretic peptide in acute ischemic stroke.

An intergroup comparison of BNP levels with the Tukey–Kramer HSD test revealed that the BNP level of the cardioembolic stroke group was significantly higher than the levels of other groups (p<0.0001) (**Fig. 2**).

**Figure 3:**
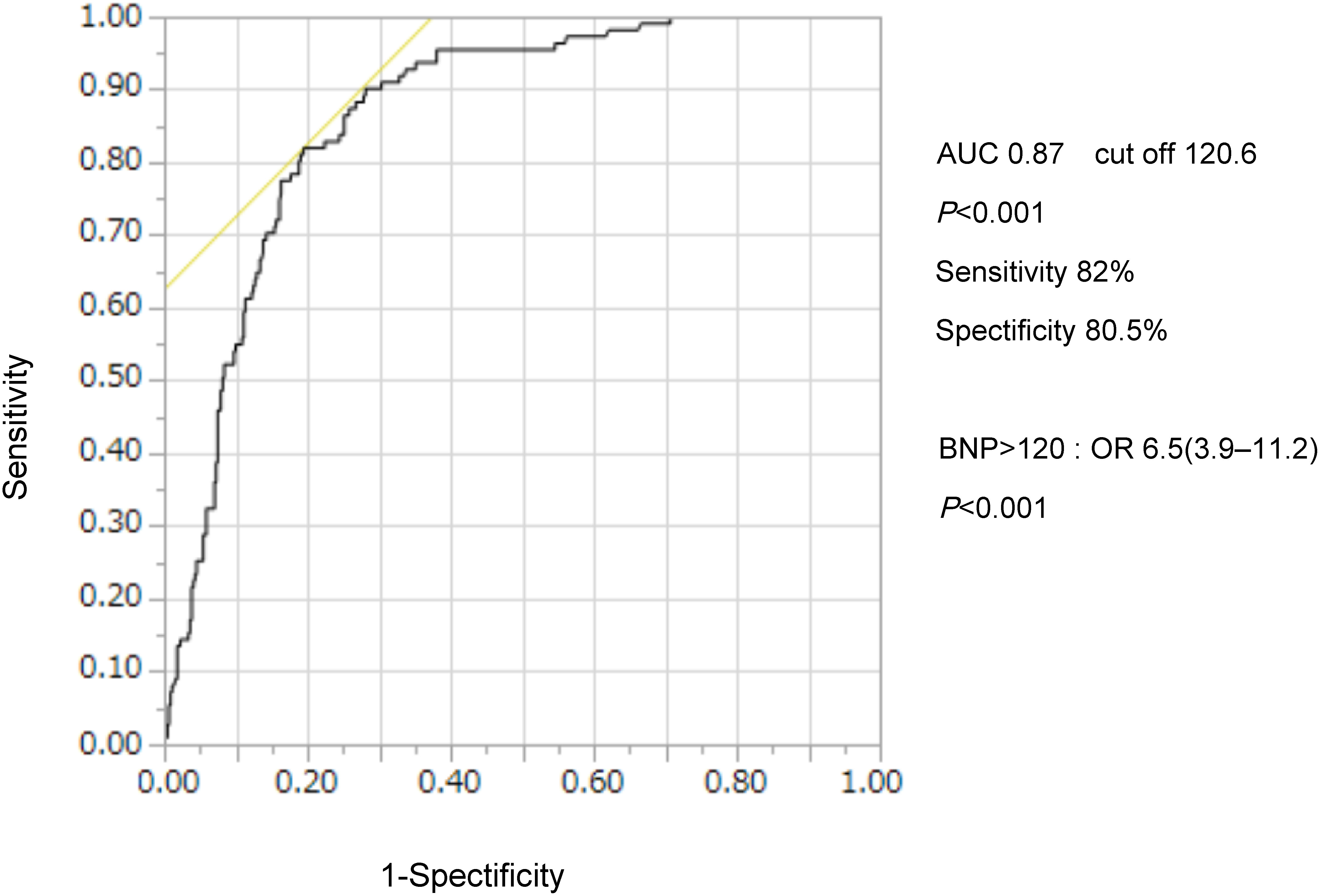
Fig. 3 ROC curve of BNP.

A univariate analysis of the BNP measurements in different subtype groups showed that BNP of ≥120 pg/mL predicted the cardioembolic subtype with an odds ratio of 6.5 (**Fig. 3**).

Results of specialized blood tests additionally conducted in patients with cerebral infarction of other determined etiology/undetermined etiology identified no hematological diseases that could facilitate embolization directly causing cerebral infarction. The thoracic-abdominal-pelvic CT and tumor marker data identified 8 cases of suspected cerebral infarction associated with malignant tumors (3 cases of lung cancer, 1 case of pancreatic cancer, 3 cases of colorectal cancer, and 1 case of malignant lymphoma). Venous ultrasonography of lower extremities identified DVT in 2 cases. Other confirmed causes of cerebral infarction included cases associated with intracranial artery dissection (7 cases; in vertebral arteries in 4 cases and anterior cerebral arteries in 3 cases), thoracic aortic dissection (1 case), aortogenic cerebral embolism (1 case), and reversible cerebral vasoconstriction syndrome (4 cases).

ICMs were inserted in 15 of 76 patients with cryptogenic stroke and detected AF in 4 cases (26%) during the observation period (223–384 days) (**Table 2**).

**Table 2:** Table 2 Summary of patients with PAF detected by ICM

Case	Age	Sex	Day of PAF detection by ICM	BNP
4	77	M	day 6	315
6	69	F	day 220	49
10	67	M	day 233	78
11	75	F	day 53	1127

**A Representative Case Study**

Case 11 (**Table 2**)

A 75-year-old woman. She was found collapsed and was rushed to a hospital.

On arrival, consciousness level: Glasgow Coma Scale E3V3M6; paralysis: left upper and lower extremities Manual Muscle Test 1/5.

Diffusion-weighted magnetic resonance imaging showed a high intensity area in the right basal ganglion. Magnetic resonance angiography did not visualize the right internal carotid artery.

**Figure 4:**
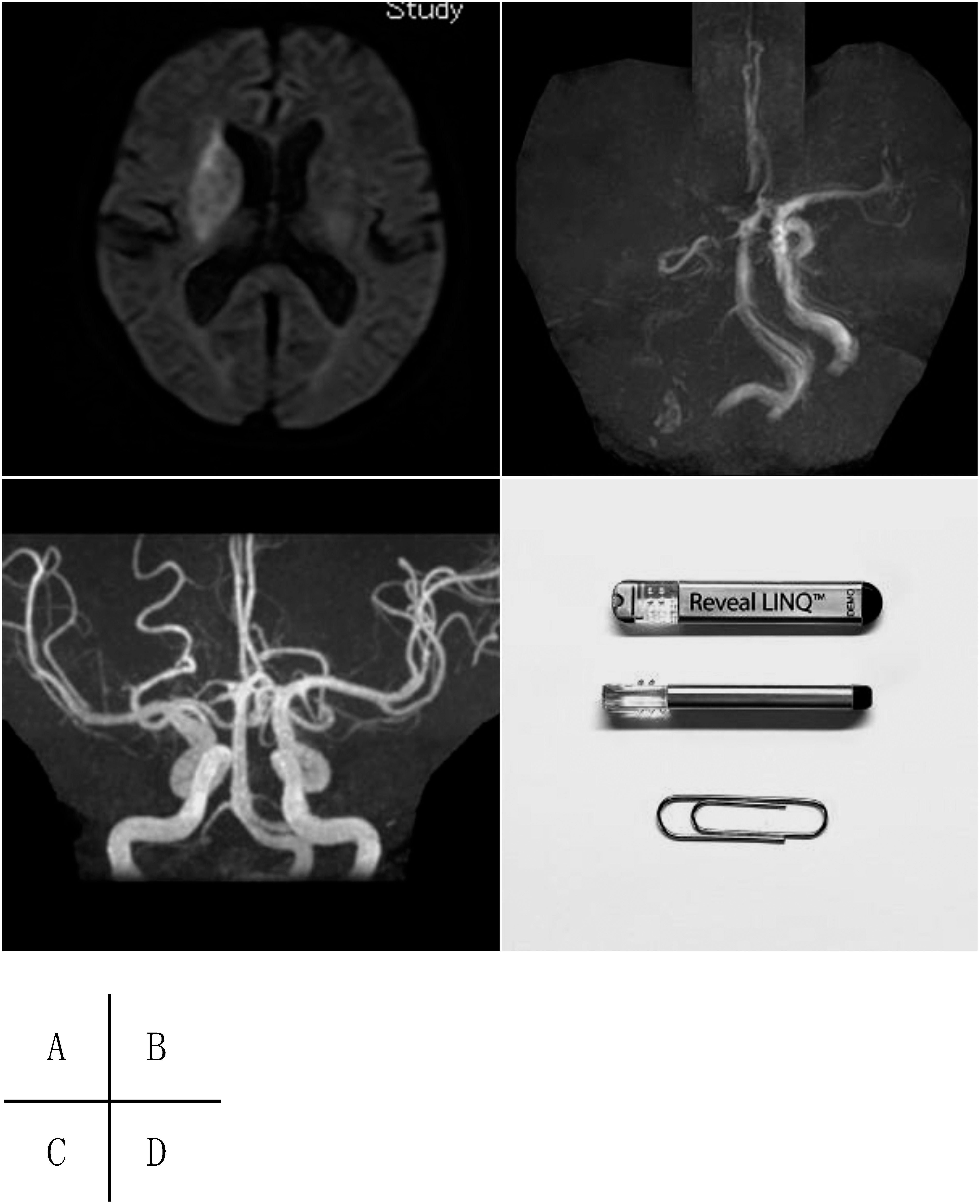
Fig. 4 A case indicating detection of PAF by ICM. (**A**) MRI (DWI) displayed a high-signal-intensity area in the right basal ganglia, however much of the MCA distribution appears spared. DWI-ASPECT 8/11. (**B**) MRA indicates right internal carotid artery occlusion. (**C**) Total recanalization after mechanical thrombectomy. No large vessel stenosis present on MRA at day 2. (**D**) ICM and paper clip. These are about the same size.

After an intravenous injection of t-PA was administered, the patient was transferred to us while receiving drip and ship treatment (**Figs. 4A** and **4B**).

On arrival, AF (−).

Since t-PA did not ameliorate symptoms, we initiated endovascular treatment. Angiography revealed T-occlusion at the right IC-top. Thrombectomy with Penumbra 5MAX ACE (penumbra, Alameda, CA, USA)+Solitire 6×40 mm (Medtronic, Dublin, Ireland) (Captive technique) resulted in TICI3 perfusion.

Angiography after thrombectomy showed no stenotic lesions in major arteries (**Fig. 4C**).

Thorough postoperative examinations including cardiac monitoring, Holter monitoring, echocardiography, and contrast-enhanced chest CT failed to identify the embolus origin; thus, an ICM was inserted under fluoroscopic guidance on day 14 to detect covert AF. While the patient was subsequently transferred to a different hospital for rehabilitation, we noted an AF alert via a remote data transmission device (Carelink [Medtronic, Dublin, Ireland]) 53 days after the ICM was inserted. We contacted the hospital to which she was transferred and requested to initiate a direct oral anticoagulant (DOAC).

**Figure 5:**
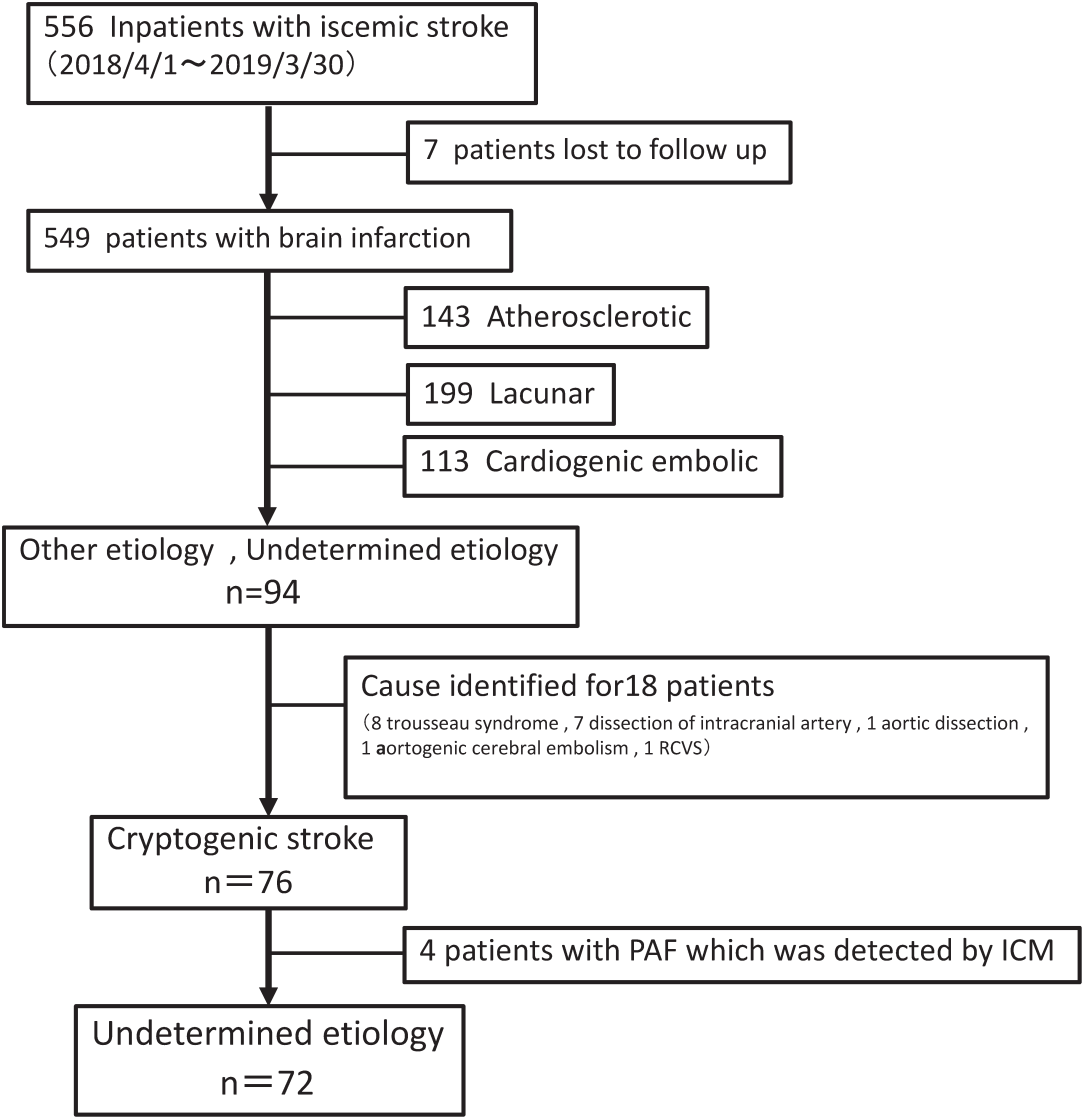
Fig. 5 Flowchart of search and screening process. According to TOAST classification, cerebral infarction of other/undetermined etiology was observed in 94 cases, of which 22 cases were found to have causes by additional workup. Implantable cardiac monitors were inserted in 15 of 76 cases of cryptogenic cerebral infarction, of which 4 cases showed detection of PAF.

Among 94 cases classified according to the TOAST criteria as “cerebral infarction of other determined etiology/undetermined etiology” at the time of hospitalization, we could identify causes in 22 cases (23.4%) through detailed examinations. Finally, a total of 72 cases of true “cerebral infarction of undetermined etiology” were noted, which accounted for 13.1% of all cases of cerebral infarction (**Fig. 5**).

## Discussion

### Importance and limitations of subtype classification

Embolic cerebral infarction is considered to account for the majority of cryptogenic strokes. Conditions underlying embolism include covert AF, paradoxical embolism caused by patent foramen ovale, presence of aortic arch plaques, and cancer. Among these conditions, detection of covert PAF is most important based on the prevalence and embolic risk. However, multiple causes can underlie, and the main cause cannot be identified in some cases.

In the present study, in addition to standard tests, we proactively used special blood tests, tumor marker tests, thoracic-abdominal-pelvic CT, and ICMs and could identify causes in 22 of 94 cases initially classified as “other determined etiology/undetermined etiology.”

However, problems in this study include the non-use of transesophageal echocardiography (TEE). TEE was not available in our hospital, which specializes in neurosurgery. TEE is excellent in the detection of patent foramen ovale (PFO), atrial septal aneurysm, and aortic arch plaques, which have been reported to be important sources of embolus in cases of cryptogenic strokes.^1)^ In this study, a thoracic-abdominal-pelvic CT examination revealed a ≥4 mm aortic arch plaque in one patient who was consequently diagnosed with aortogenic embolic stroke, suggesting the possibility that sources of emboli may have been overlooked owing to non-use of TEE. The detection of PFO and DVT by TEE and venous ultrasonography of lower extremities, respectively, indicates a high likelihood of paradoxical cerebral embolism, allowing for appropriate secondary stroke prevention. In Japan, PFO closure as a recurrence prevention measure for cryptogenic strokes with PFO has been included in insurance coverage since December 2019.

Although mechanical thrombectomy is currently performed in more than 700 institutions, TEE remains difficult in many small- and medium-sized hospitals, similar to ours, specializing in neurosurgery. However, TEE will become essential, owing to the fact that its effectiveness for the detection of embolus sources has been demonstrated in an increasing number of reports.

Examinations alternative to TEE include transcranial doppler (TCD) ultrasonography, transcranial color flow imaging (TC-CFI), and contrast-enhanced CT of the left atrial appendage. TCD and TC-CFI are useful tests for patients capable of obeying instructions and undergoing the Valsalva maneuver (breath-holding), since right-to-left intracardiac shunts can be identified as high intensity transient signals.^2)^ Bilchick et al. have reported that contrast-enhanced CT of the left atrial appendage can be used to detect detection left atrial appendage thrombi in a minimally invasive manner.^3)^

### Trousseau’s syndrome

Patients suspected of having “cerebral infarction due to hypercoagulation associated with malignant tumors” were diagnosed as Trousseau’s syndrome. Adenocarcinomas such as lung cancer, pancreatic cancer, gastric cancer, and ovarian cancer (mucin-producing tumors) have been reported to commonly underlie Trousseau’s syndrome.^4)^ As reported previously, imaging examinations showed bilateral, scattered multiple emboli in the vast majority of cerebral infarction cases. In one case, however, a major artery (left middle cerebral artery) was occluded, and thrombectomy was performed. Furthermore, this treatment is important for survival improvement and recurrence prevention owing to the fact that malignant tumors are discovered following the onset of cerebral infarction. However, no consensus about secondary prevention, other than the treatment of underlying tumors, has been established. The patients underwent management with heparin during hospitalization and anticoagulant therapy with warfarin after discharge, before they were referred to specialists.

### Significance of BNP measurement in subtyping

BNP, which is used for screening for heart disorders and heart failure, has recently been reported to be an important auxiliary diagnostic marker for subtyping cerebral infarction. Shibazaki et al. have stated that a cut-off BNP level of 140 pg/mL could be used to identify cardiogenic embolism with a sensitivity of 80.5% and a specificity of 80.5%.^5)^ Reportedly, BNP-65 pg/mL was independently associated with covert AF and the detection rate of new-onset AF increased with the BNP level.^6)^ BNP measurements are considered to require some correction for age because they correlate positively with age. However, no established correction methods are currently available.

### Breakdown of cardiogenic embolisms and the effectiveness of ICMs

PAF has been reported to account for approximately 35%–40% of cardiogenic embolism cases; however, the detection of PAF is not easy. A previous study has reported that cardiac monitoring for at least 72 h is required to detect PAF and AF in patients with cryptogenic stroke.^7)^ Higgins et al. have reported the effectiveness of wearable cardiac monitor use for 1 week for the detection of PAF,^8)^ and our results in this study also suggest the necessity of continuous monitoring for at least 3–7 days after hospitalization. Furthermore, the ICM use for PAF detection in patients with cryptogenic cerebral infarction was added to the insurance coverage in September 2017 in Japan, since a longer duration of monitoring facilitates the detection of covert AF. In this study, we proactively encouraged patients with cryptogenic stroke to use ICMs, and detected 4 cases of AF (26%) during the follow-up period in 15 patients who consented to wear Reveal LINQ (Medtronic, Dublin, Ireland) (**Fig. 4D**). This detection rate is clearly higher than 12.4%/year in the CRYSTAL-AF study.

The detection rate is expected to be even higher when the analysis includes only the cases in which thrombectomy resulted in complete recanalization. The 30% AF detection during the ICM use for 36 months in the CRYSTAL-AF study^9)^ suggests that the need for continued careful monitoring is imperative.

### Treatment of cryptogenic stroke/ESUS

Contrary to expectations, results of two large clinical studies for addition of ESUS to indications of DOACs (NAVIGATE ESUS and RE-SPECT ESUS) did not demonstrate that DOACs were safer and more effective than aspirin.^10,11)^ Currently, DOACs should not be used for treatment of cerebral infarction of undetermined etiology without careful consideration. Nevertheless, detection of PAF and initiation of evidence-based anticoagulant therapy as early as possible are necessary because recurrence of cardiogenic embolism is likely to have serious consequences.

## Conclusion

Thrombectomy has been established as an effective treatment for cerebral infarction owing to acute major artery occlusion; however, sources of emboli and causative conditions are not necessarily explored fully for after endovascular treatment at all institutions. Determination of etiology of cerebral infarction is critical because appropriate treatment for prevention of recurrence is possible only after accurate disease subtyping. BNP measurement, tumor markers, thoracic-abdominal-pelvic CT, 1-week Holter cardiac monitoring, and ICMs were useful for determination of cerebral infarction subtypes and detection of covert AF at institutions specializing in neurosurgery. Meanwhile, thrombohemostatic blood tests did not appear to be effective.

## Notes

This is secondary publication from J Jpn Coll Angiol 2021; 61: 49–55.
